# Using Corticomuscular Coherence to Reflect Function Recovery of Paretic Upper Limb after Stroke: A Case Study

**DOI:** 10.3389/fneur.2017.00728

**Published:** 2018-01-10

**Authors:** Yang Zheng, Yu Peng, Guanghua Xu, Long Li, Jue Wang

**Affiliations:** ^1^State Key Laboratory for Manufacturing Systems Engineering, School of Mechanical Engineering, Xi’an Jiaotong University, Xi’an, China; ^2^The Key Laboratory of Biomedical Information Engineering of Ministry of Education, School of Life Science and Technology, Institute of Biomedical Engineering, Xi’an Jiaotong University, Xi’an, China; ^3^The Department of Rehabilitation Medicine, First Affiliated Hospital of Xi’an Jiaotong University, Xi’an, China

**Keywords:** stroke, electromyography, electroencephalography, corticomuscular coherence, motor impairment

## Abstract

**Purpose:**

Motor deficits after stroke are supposed to arise from the reduced neural drive from the brain to muscles. This study aimed to demonstrate the feasibility of reflecting the motor function improvement after stroke with the measurement of corticomuscular coherence (CMC) in an individual subject.

**Method:**

A stroke patient was recruited to participate in an experiment before and after the function recovery of his paretic upper limb, respectively. An elbow flexion task with a constant muscle contraction level was involved in the experiment. Electromyography and electroencephalography signals were recorded simultaneously to estimate the CMC. The non-parameter statistical analysis was used to test the significance of CMC differences between the first and second times of experiments.

**Result:**

The strongest corticomuscular coupling emerged at the motor cortex contralateral to the contracting muscles for both the affected and unaffected limbs. The strength of the corticomuscular coupling between activities from the paretic limb muscles and the contralateral motor cortex for the second time of experiment increased significantly compared with that for the first time. However, the CMC of the unaffected limb had no significant changes between two times of experiments.

**Conclusion:**

The results demonstrated that the increased corticomuscular coupling strength resulted from the motor function restoration of the paretic limb. The measure of CMC can reflect the recovery of motor function after stroke by quantifying interactions between activities from the motor cortex and controlled muscles.

## Introduction

Stroke is one of the major diseases that cause long-term motor deficits of adults (1). However, our poor understanding of the mechanisms underlying motor impairments after stroke limits greatly the development of effective intervention and evaluation methods. In general, motor impairments after stroke are deemed to arise from changes in both neural and muscle properties. Poststroke changes in the neural system have been studied from different points of view such as the decreased excitability of the affected cortex (2, 3) and the increased inhibitory effect from the unaffected hemisphere on the affected hemisphere (4). Spasm and flaccid paresis of muscles are believed to result from the loss of control input from the brain at different phases after stroke. Even though stroke survivors have been demonstrated to have significant descending information flow in the affected side during the chronic period (5), there is evidence that poststroke impairments reflect the reduced central neural drive to muscles. Mima et al. and Fang et al. found that the functional coupling between cortical commands and consequent muscle activities of stroke subjects were weaker than that of healthy controls (6, 7). The conduction time from the central cortical rhythm to peripheral oscillations in the affected side was significantly prolonged compared with that of the unaffected side after stroke (8).

It is believed that stroke interrupts the motor-related neural network and then reduces the neural drive to the muscles. The coherent activities between the motor cortex and the muscles are believed to reflect the synchronized discharge of corticospinal cells (9). It can be estimated by analyzing the frequency domain coherence (10) between electromyography (EMG) and electroencephalography (EEG) signals termed as corticomuscular coherence (CMC). Although previous studies have demonstrated that the CMC strength of poststroke subjects was weaker than that of healthy controls, it is still not clear whether the corticomuscular coupling will enhance along with the motor function recovery to directly reflect the motor function state of paretic limbs after stroke. In the current study, a poststroke patient was recruited to participate in two times of experiments involving an elbow flexion task. The time interval between two times of experiments was determined to guarantee that the patient had obtained an obvious motor function recovery of the affected upper extremity. CMC from two times of experiments was estimated and compared to verify whether motor function recovery can be reflected by the change of corticomuscular coupling strength.

## Backgrounds

### Experiment and Subject

**Figure 1:**
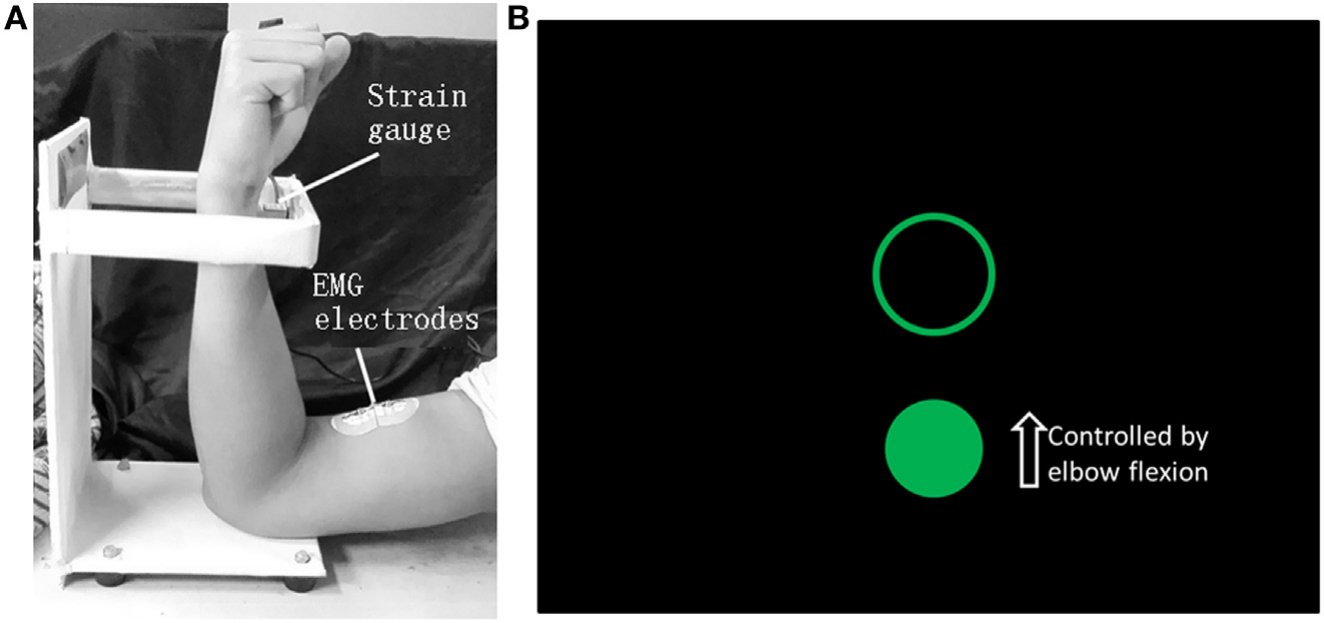
The motion task of elbow flexion **(A)** and the visual feedback information on screen **(B)**. When the biceps brachii contracts, the wrist will press the strain gage and the force level can be detected. The circle can be shifted vertically by applying force to the strain gage and the position of the ring is fixed. The subject was requested to move the circle into the ring as soon as possible when a trial started and maintain the force until the end of a trial when the circle and the ring both disappeared. The force needed to shift the circle into the ring was 3 N.

An elbow flexion task was designed for the stroke patient because only poor rehabilitation outcomes can be generally obtained for hand. The force applied by the elbow flexion was monitored by a strain gage and fed back to the patient visually to help him finish the task with moderate and constant muscle contractions (11), because coherence analyses (12, 13) have demonstrated that the coupling is most pronounced in the beta-band range during steady muscle contractions and the beta-band CMC is assumed to be associated with strategies for controlling submaximal muscle forces (12, 14, 15). The designed motion task and the visual feedback information on screen are illustrated in Figures 1A,B, respectively. A trial was initiated when a circle and a target ring showed on screen and was over when they disappeared. Each trial lasted 11 s and there was a 2-s long interval between adjacent trials. Each run contained 20 trials and each side of upper limbs performed two runs, respectively. The subject practiced before data recording until the target force could be reached within the first 2 s of each trial.

The Net Amps 300 system together with a polygraph input box (Electrical Geodesics Inc.) was selected to amplify the EEG and EMG signals. An elastic cap that has 128 electrodes was used to detect EEG activities, and EMG signals were recorded from the biceps brachii using two adhesive surface electrodes with a 2-cm interelectrode distance. The reference electrode for EEG recording was located at the “Cz” position. The ground electrode, common for EEG and EMG signals, was positioned on the midline of the scalp at the level of the prefrontal cortex. EEG and EMG signals were sampled at 1,000 Hz together with event markers to synchronize with force signals. Force data were recorded simultaneously from the strain gage at a sampling rate of 200 Hz. All signals were saved on a hard disk for off-line analyses.

The subject suffered from his first cerebral hemorrhage at the right basal ganglia and had paralysis of his left upper extremity. His age was 35. No cognition impairment was reported. Two times of experiments were performed with an interval of 1 month. The first time was carried out 6 weeks after the stroke onset. During the period, the subject was in hospital and received regular rehabilitation trainings including physical therapy and occupational therapy. The Fugl-Meyer (upper limb section) score was 15 points for the first time of experiment and a Fugl-Meyer score of 38 points was obtained for the second time. The subject participated in the experiment with the approval of the local ethics committee and the informed consent in accordance with the Declaration of Helsinki. The written informed consent was obtained from the subject for the publication of this case report.

### Data Analyses

The same CMC estimation method was used as in our previous study (11). Original EEG and EMG signals were first filtered using a two-pass finite impulse response filter with a pass band from 3 to 45 Hz. Artifacts caused by eye blinks, cardiac activities, and power-line interferences were eliminated with the independent component analysis method (16, 17). Then, EEG and EMG signals within the muscle contraction period of each trial were segmented to 1 s long segments with no overlap. EEG signals from all electrodes within each segment were transformed into the reference-free current source density (CSD) distribution using the scalp surface Laplacian (18–20). The CMC spectrum for each EEG electrode was determined by calculating the coherence between the corresponding CSD signal and the unrectified EMG signal with the multitaper method (21).

A non-parameter statistic method (22) was used to compare the difference of the CMC between the first and second times of experiments. This method can solve the unequal estimation bias problem and the multiple comparison problem in the statistical analysis of frequency domain coherence differences under two different conditions (22). The original CMC difference *C*_1_(*f*) − *C*_2_(*f*) was transformed to the z-spectrum CMC difference,

$$Z\left(f\right)=\frac{\left({\text{tanh}}^{-1}\left(C_{1}\left(f\right)\right)-n_{1}\right)-\left({\text{tanh}}^{-1}\left(C_{2}\left(f\right)\right)-n_{2}\right)}{\sqrt{n_{1}+n_{2}}},$$

where *n*_1_ = 1/*d*_1_ − 2, *n*_2_ = 1/*d*_2_ − 2, *C*_1_(*f*) and *C*_2_(*f*) are the CMC spectrum of condition 1 and 2, respectively. *d*_1_ and *d*_2_ are the number of degrees of freedom for coherence estimation (22). The procedure to estimate the *p-*value included several steps. Data segments from both conditions were first merged into a single set and then randomly selected to construct new data sets for both conditions. The result of this procedure was called a random partition and the corresponding *Z*(*f*) was estimated. A test statistic that was based on the clustering of adjacent frequencies (22) was extracted from *Z*(*f*) to avoid the multiple comparison problem. The procedures above were repeated for a large number of times (5,000 times), and a histogram of the test statistics from all random partitions was constructed. From the test statistic calculated with the actually observed data and the histogram constructed with all random partitions, the proportion of the random partitions that resulted in a larger test statistic than the observed one, i.e., the Monte Carlo *p* value, was calculated.

### Results

**Figure 2:**
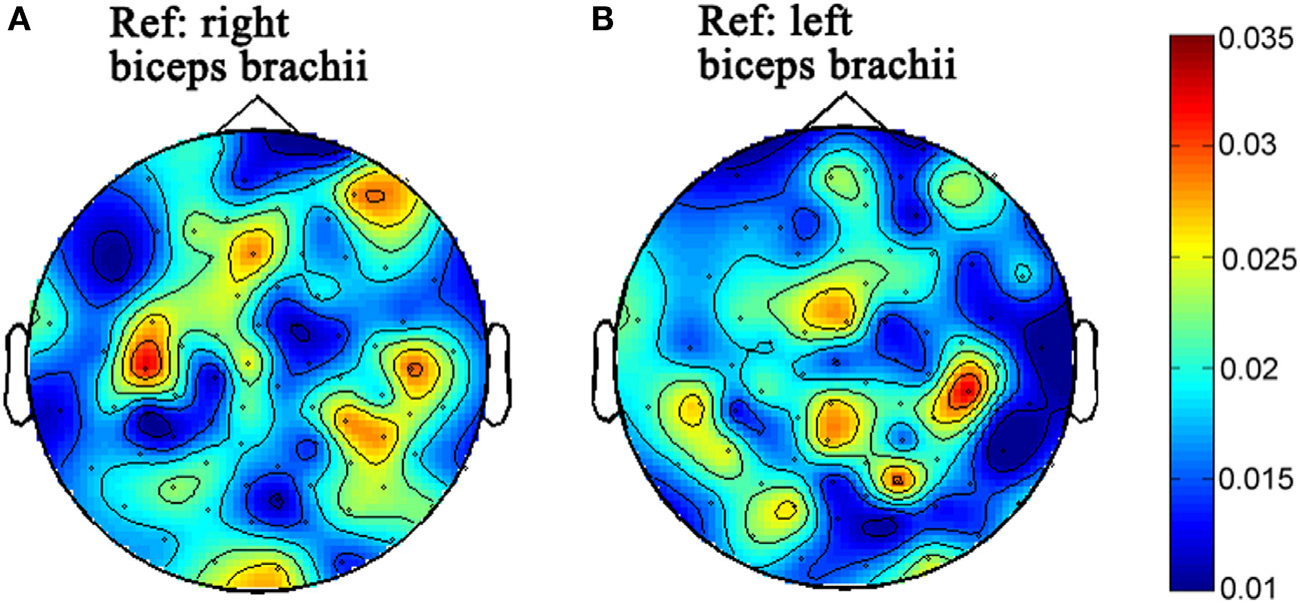
The topographies of beta-band (15–30 Hz) corticomuscular coherence with the peripheral reference of right biceps brachii **(A)** and left biceps brachii **(B)**, respectively for the first time of experiment.

**Figure 3:**
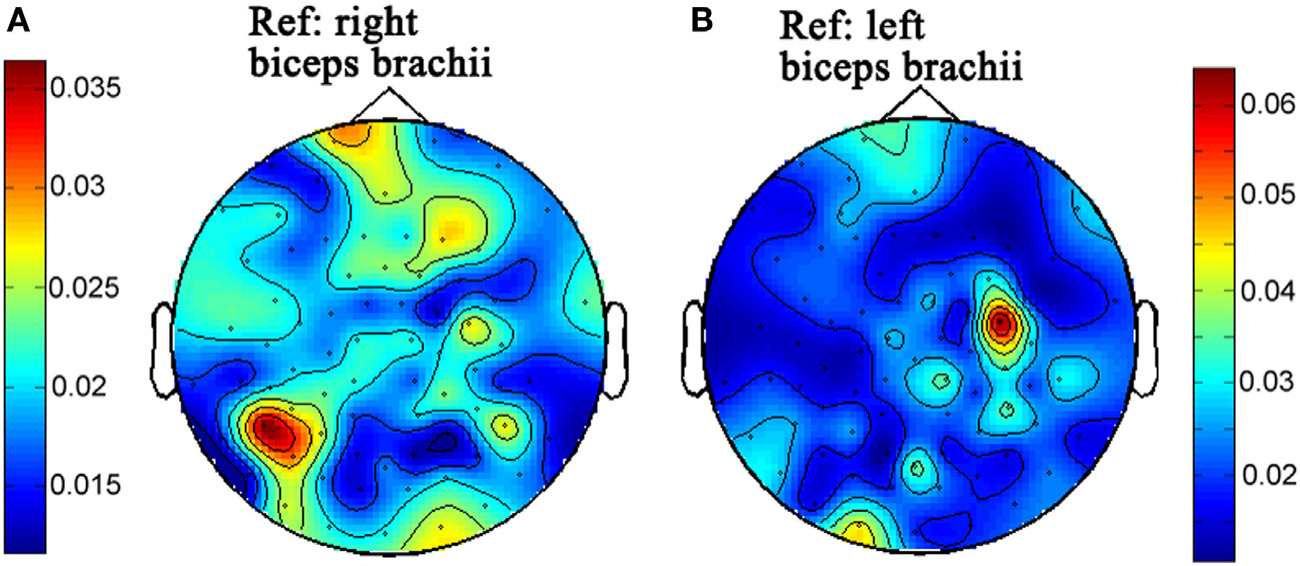
The topographies of beta-band (15–30 Hz) corticomuscular coherence with the peripheral reference of right biceps brachii **(A)** and left biceps brachii **(B)**, respectively for the second time of experiment.

The topographies of beta-band (15–30) CMC from the first and second times of experiments are illustrated in Figures 2 and 3, respectively. Figures 2A and 3A illustrate the CMC topographies between activities from the cortex and the right biceps brachii. Figures 2B and 3B show the results with the left biceps brachii as the peripheral reference. A consistent result was that the strongest CMC emerged at the contralateral motor area to the contracting muscles. Meanwhile, there were minor differences about the electrode locations where the strongest CMC showed up between the first and second times of experiments. They might result from the small shift of wearing positions of the electrode cap. Further statistical analyses were based on the CMC spectrum obtained at the electrodes where the strongest beta-band corticomuscular coupling emerged.

**Figure 4:**
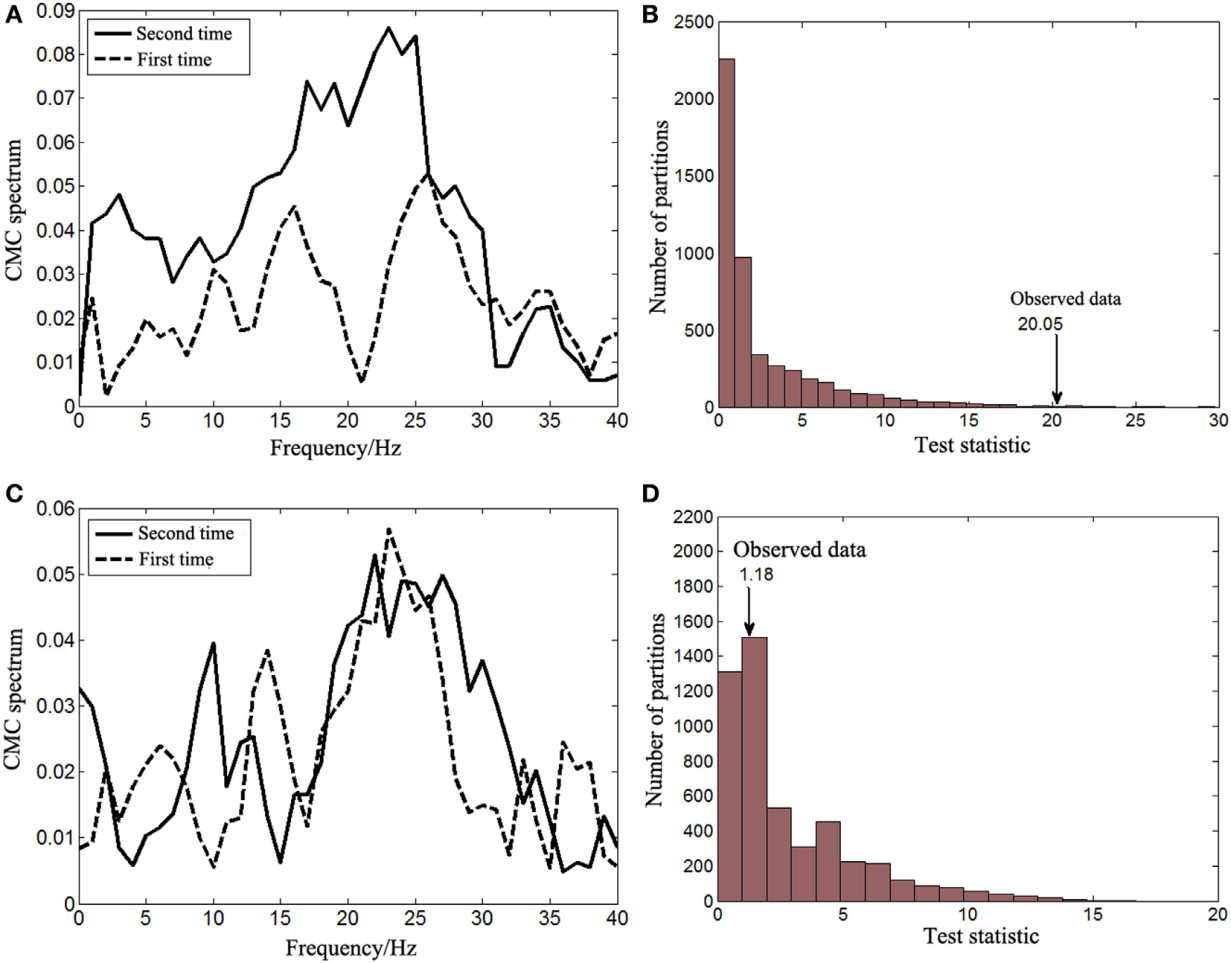
The corticomuscular coherence (CMC) spectrum between activities from the right motor cortex and the left biceps brachii **(A)** and the results of the corresponding statistical analysis for the beta-band CMC differences between two times of experiments **(B)**. The CMC spectrum between activities from the left motor cortex and the right biceps brachii **(C)** and the results of the corresponding statistical analysis for the beta-band CMC differences between two times of experiments **(D)**.

The non-parameter statistical analysis was performed to verify whether there were significant differences of the beta-band CMC between the first and second times of experiments. The CMC spectrum between activities from the left biceps brachii (affected side) and the contralateral motor cortex is illustrated in Figure 4A. For the first time of experiment, the spectrum peaks within the beta band were located at 16 and 26 Hz, respectively and there was a sharp decrease at 21 Hz. For the second time of experiment, the spectrum peaks were similarly located within the beta band at around 18 and 23 Hz. The beta-band CMC magnitude of the second time was apparently larger than that of the first time. The null hypothesis was that the beta-band CMC of the second time was not larger than that of the first time when muscles of the affected side contracted. The distribution of test statistics from 5,000 random partitions is shown in Figure 4B. The test statistic of the observed data was 20.05 and the resulting Monte Carlo *p*-value was 0.005. Thus, the null hypothesis was rejected and the beta-band CMC of the second time was significantly larger than that of the first time when muscles of the affected side contracted.

The significance of the beta-band CMC difference between the first and second times of experiments when the right biceps brachii (unaffected side) contracted was also tested with the non-parameter statistical analysis. The CMC spectrum of both times of experiments is illustrated in Figure 4C. There was no obvious difference of the CMC spectrum within the beta band. The peaks were both located at approximately 24 Hz and their amplitudes were similar. The null hypothesis was, therefore, set to be that there was no significant difference of the beta-band CMC between the first and second times of experiments when muscles of the unaffected side contracted. The distribution of test statistics from 5,000 random partitions is shown in Figure 4D, and the test statistic of the observed data was 1.18 with a Monte Carlo *p-*value of 0.7306. The null hypothesis cannot be rejected and, therefore, it was correct.

## Discussions

Poststroke motor deficits arise essentially from the damages to the descending spinal tracks. The damage makes the brain lose control of limb muscles. CMC is believed to be a direct measure of interactions between physiological oscillatory activities from the brain and the controlled muscles. Therefore, the corticomuscular coupling strength might be weakened by stroke theoretically. However, it is not known whether stroke survivors can regain the normal strength level of corticomuscular coupling after motor function recovery. The most significant result of the current study was that the corticomuscular coupling strength between activities from the paretic limb muscles and the contralateral motor cortex increased along with the recovery of the patient’s paretic limb. However, the CMC of the unaffected limb had no significant changes, which demonstrated that the familiarization with the task could not contribute to the increase of the corticomuscular coupling strength in the current study. Therefore, the increased coupling strength of the affected limb would only result from the motor function recovery of the paretic upper limb. This means that the measure of CMC can reflect the change of abilities for the brain to control muscular activities after stroke. Further studies will recruit more stroke subjects and focus on the sensitivity of the CMC as a measure to quantify the motor function state of paretic limbs.

The corticomuscular coupling is weakened for poststroke patients compared with healthy controls (6, 7). The results in the current study further demonstrated that the recovery of motor function was related to the enhanced corticomuscular coupling. In addition, previous studies showed that improved fine movement control was related to higher CMC (13, 23) and motor learning was associated with increased CMC and better performance (23). Thus, it may be speculated that increasing the corticomuscular coupling strength of the paretic limb for stroke patients is both the goal and the way for regaining motor function. Many factors have been identified to influence the corticomuscular coupling strength and some of them have the potential to be utilized in limb rehabilitation interventions (11). Above all, the results of the current study remind us that exploring the influence of rehabilitation interventions on corticomuscular coupling may provide another way to verify their validity from a new point.

In the current study, the CMC showed significant beta-band differences. This was inconsistent with Fang’s study in which CMC differences between stroke patients and healthy controls mainly occurred in the gamma band (6). This might be caused by the fact that different motion tasks were used. The elbow flexion task in the current study involved isometric muscle contractions with static output force while the reaching task in Fang’s study was more related to isometric muscle contractions with dynamic output force. Omlor et al. (24) demonstrated that corticomuscular coupling occurred within the beta band under the condition of isometric muscle contractions with static output force; whereas the most significant CMC shifted to the gamma band with isometric muscle contractions with dynamic output force. The difference of coherent frequency bands might reflect different ways for the brain to control muscle contractions under different conditions.

The CMC spectrum in Figure 4 showed that the peak magnitude of beta-band CMC of the affected side was larger than that of the unaffected side after recovery. This is consistent with the results from Mima’s study in which stroke survivors at their chronic phase were recruited (7). The study demonstrated that the corticomuscular coupling of unaffected side was significantly stronger than that of affected side during power grip and wrist extension. However, the corticomuscular coupling of unaffected side was slightly weaker during elbow flexion compared with that of affected side even though it is counterintuitive. The exact underlying mechanism is not known but might be associated with the contribution of the descending direct corticospinal pathway to the proximal and distal muscles after stroke (25). Further studies will be needed to verify its universality and fully understand the neural mechanism.

The limitation of the current study was that we cannot tell whether the increased corticomuscular coupling strength resulted from spontaneous recovery or motor rehabilitation training. Since the patient was at his subacute stage of stroke, spontaneous recovery could happen. Meanwhile, he received physical and occupational therapy that also might result in his recovery. Therefore, it cannot be verified through our study whether the spontaneous recovery or the rehabilitation training alone, or both contributed to the increased corticomuscular coupling. This will be explored in our further studies.

## Concluding Remarks

This study demonstrated that the corticomuscular coupling strength for the paretic limb increased along with its function recovery while the coupling strength for the unaffected limb did not change through a case study. We concluded that interrupted corticomuscular interactions account for the motor deficits and CMC, as a measure of coherent activities between motor cortex and controlled muscles, can reflect the function recovery of paretic limbs after stroke.

## Ethics Statement

This study was carried out in accordance with the recommendations of the Biomedical Research Ethics Committee of School of Life Science and Technology in Xi’an Jiaotong University with written informed consent from the subject. The subject gave written informed consent in accordance with the Declaration of Helsinki. The protocol was approved by the Biomedical Research Ethics Committee of School of Life Science and Technology in Xi’an Jiaotong University.

## Author Contributions

Drafted manuscript and contributed to acquisition, analysis, and interpretation: YZ. Critically revised the manuscript for important intellectual content and gave final approval and agreement to be accountable for all aspects of the work: YZ, YP, GX, LL, and JW. Contributed to conception or design: YZ, YP, and LL. Contributed to conception and interpretation: GX and JW. Contributed to acquisition and interpretation: YP and LL.

## Conflict of Interest Statement

The authors declare that the research was conducted in the absence of any commercial or financial relationships that could be construed as a potential conflict of interest.
